# Phenotypic profiling of human induced regulatory T cells at early differentiation: insights into distinct immunosuppressive potential

**DOI:** 10.1007/s00018-024-05429-3

**Published:** 2024-09-12

**Authors:** Roosa Kattelus, Inna Starskaia, Markus Lindén, Kedar Batkulwar, Sami Pietilä, Robert Moulder, Alexander Marson, Omid Rasool, Tomi Suomi, Laura L. Elo, Riitta Lahesmaa, Tanja Buchacher

**Affiliations:** 1 Turku Bioscience Centre, University of Turku and Åbo Akademi University, Turku, Finland; 2 InFLAMES Research Flagship Center, University of Turku, Turku, Finland; 3 Turku Doctoral Programme of Molecular Medicine, University of Turku, Turku, Finland; 4 Gladstone-UCSF Institute of Genomic Immunology, San Francisco, CA 94158 USA; 5 Department of Medicine, University of California San Francisco, San Francisco, CA 94143 USA; 6 Institute of Biomedicine, University of Turku, Turku, Finland

**Keywords:** Regulatory T cells, Differentiation, FOXP3, CD103, Mass cytometry, Mass spectrometry

## Abstract

Regulatory T cells (Tregs) play a key role in suppressing systemic effector immune responses, thereby preventing autoimmune diseases but also potentially contributing to tumor progression. Thus, there is great interest in clinically manipulating Tregs, but the precise mechanisms governing in vitro-induced Treg (iTreg) differentiation are not yet fully understood. Here, we used multiparametric mass cytometry to phenotypically profile human iTregs during the early stages of in vitro differentiation at single-cell level. A panel of 25 metal-conjugated antibodies specific to markers associated with human Tregs was used to characterize these immunomodulatory cells. We found that iTregs highly express the transcription factor FOXP3, as well as characteristic Treg-associated surface markers (e.g. CD25, PD1, CD137, CCR4, CCR7, CXCR3, and CD103). Expression of co-inhibitory factors (e.g. TIM3, LAG3, and TIGIT) increased slightly at late stages of iTreg differentiation. Further, CD103 was upregulated on a subpopulation of iTregs with greater suppressive capacity than their CD103^−^ counterparts. Using mass-spectrometry-based proteomics, we showed that sorted CD103^+^ iTregs express factors associated with immunosuppression. Overall, our study highlights that during early stages of differentiation, iTregs resemble memory-like Treg features with immunosuppressive activity, and provides opportunities for further investigation into the molecular mechanisms underlying Treg function.

**Supplementary Information:**

The online version contains supplementary material available at 10.1007/s00018-024-05429-3.

## Introduction

Regulatory T cells (Tregs) are an essential immunomodulatory subset of the human immune system and constitute 5–10% of the peripheral blood CD4^+^ T cells [1, 2]. Tregs suppress excessive effector T cell responses to ensure peripheral self-tolerance and immune homeostasis [3]. Thus, abnormalities in Treg numbers, frequencies, and suppressive function are potentially associated with autoimmune pathology, whereas Treg accumulation in tumors suppresses anti-tumor immunity [4, 5].

Most Tregs are produced by the thymus or extrathymically in the periphery from conventional naïve CD4^+^ T cells upon antigen exposure. In addition, Tregs can also be induced from naïve CD4^+^ T cells in vitro (iTreg) upon T cell receptor (TCR) signaling in the presence of cytokines [6, 7]. The transcriptional signature of Tregs is controlled by the lineage-specifying transcription factor (TF) FOXP3. FOXP3 is essential for the development and immunosuppressive function of Tregs [8]. Besides FOXP3, other TFs (e.g. HIC1) are associated with human Treg differentiation [7, 9].

Additionally, Tregs express multiple co-stimulatory, co-inhibitory and other surface molecules that allow them to migrate to inflammatory sites while others function as immune checkpoints regulating immunity in both healthy and disease states. Co-inhibitory molecules, including PD1 and CTLA4, that directly suppress T cell function, have been targeted in anti-cancer therapy [5, 10]. Moreover, Tregs expressing the alpha chain of the integrin αEβ7 (CD103) have an increased ability to migrate to and be retained in peripheral tissues, and to sites of acute inflammation in murine models [11, 12], and possess a more active suppressive phenotype compared to CD103^−^ Tregs [13]. Expression of CD103 on human Tregs is limited to a minor subset [14], and its significance in the initial stages of iTreg differentiation has not been extensively investigated.

The manipulation of Tregs is a promising strategy for treating various human diseases, including autoimmunity [15, 16], transplant rejection [17] and cancer [18]. Despite their importance to human diseases, the exact molecular mechanisms regulating human iTreg development and functions are poorly understood.

In the current study, we applied multiparametric mass cytometry analysis to phenotypically profile human iTregs during early stages of in vitro differentiation. A panel of 25 metal-conjugated antibodies, specific for markers associated with human Tregs, was used to characterize these immunomodulatory cells at a high-dimensional single-cell analysis. We found that iTregs express FOXP3 along with characteristic Treg-associated surface markers (e.g. CD25, PD1, CD137, CCR4, CCR7, CXCR3 and CD103). Levels of co-inhibitory factors (e.g. TIM3, LAG3, and TIGIT) increased slightly at late stages of iTreg differentiation. CD103 was selectively expressed on a subpopulation of iTregs with a greater suppressive capacity than their CD103^−^ counterparts. Further, CD103 expression positively correlated with pro-immunosuppressive modulators. Thus, during the initial phases of differentiation, iTregs exhibit characteristics similar to memory-like Tregs with immunoregulatory activity. Our study opens possibilities for studying molecular mechanisms of Treg function in health and disease.

## Materials and methods

### CD4^+^CD25^−^ T cell isolation and differentiation

We used naïve CD4^+^ T cells isolated from umbilical cord blood, because compared to those from adult blood, they provide a purer, less-exposed model of naïve T cells with greater proliferative capacity and plasticity, making them ideal for studying early stages of iTreg differentiation, development, and therapeutic interventions. Primary human CD4^+^ T cell isolation and iTreg differentiation were performed as described earlier [9]. In brief, CD4^+^ T cells were isolated from umbilical cord blood of healthy neonates (Turku University Central Hospital, Turku, Finland) using the Ficoll-Paque (Cytiva, Cat# 17144003) density gradient centrifugation. CD4^+^ T cells were further enriched using CD4^+^ Dynal positive selection beads (Invitrogen, Cat# 11331D). CD25^+^ T cells were depleted using the CD25 Microbeads II kit (Miltenyi Biotec, Cat# 130-092-983) according to the manufacturer´s instructions. Naïve CD4^+^CD25^-^ T cells, which were highly positive for CD45RA and negative for CD45RO (Fig. S1), were activated with plate-bound anti-CD3 (500 ng/ml, Beckman Coulter Cat# IM1304; RRID: AB_131612) and soluble anti-CD28 (500 ng/ml, Beckman Coulter, Cat# IM1376; RRID: AB_131624) in X-vivo 15 medium (Lonza) supplemented with L-glutamine (2 mM), penicillin (50 U) and streptomycin (50 µg/ml) (all from Biowest) (X-Vivo medium complete). Induction of iTreg cell differentiation was done in the presence of transforming growth factor-β (TGFβ) (10 ng/ml; R&D Systems, Cat# 240-B), IL2 (12 ng/ml; R&D Systems, Cat# 202-IL-010), all-trans retinoic acid (ATRA) (10 nM; Sigma-Aldrich), and human serum (10%,Biowest Cat# S4190C). As controls, naïve CD4^+^ T cells were activated with anti-CD3 and anti-CD28, but without cytokines (Th0). The iTreg and Th0 control cells were cultured for 72 h.

To study CD103 induction during iTreg differentiation, naïve CD4^+^ T cells were cultured under the following conditions: CD3/CD28 activation (Th0), Th0 with ATRA, Th0 with IL2, Th0 with TGFβ, Th0 with ATRA and TGFβ, and Th0 with IL2 and TGFβ for 72 h. Concentrations of these factors were as described for Treg culture conditions. The protein level of CD103 was estimated by flow cytometry at 72 h of differentiation.

### CRISPR-Cas9-mediated FOXP3 ablation

Guide RNAs (gRNAs) were assembled in vitro with the *Cas9* protein as described [19]. Briefly, crisprRNA (crRNA)s, designed using Synthego gRNA design tool and synthesized by Integrated DNA Technologies (IDT), and tracrRNA (Alt-R CRISPR-Cas9 tracrRNA, Cat# 1072533, IDT) were reconstituted to 160 µM and combined in equimolar amounts (1:1), followed by incubation at 37^o^C for 30 min to prepare 80 µM gRNA reagent. Assembled gRNA was then mixed with equal volume of 40 µM recombinant *S. pyogenes* Cas9-nuclear localization sequence (NLS) purified protein (QB3 MacroLab, University of California, Berkeley) (giving 2:1 gRNA to Cas9 molar ratio) together with 1 µl of 100 µM non-homologous single-strand DNA enhancer (ssODNenh), synthesized by IDT, and incubated for 10 min at 37 °C for a final concentration of 20 µM CRISPR-Cas9 ribonucleoprotein (RNP). For FOXP3 ablation, a pool of FOXP3-targeting gRNAs (5´-TCTTCGACTTCATCACGGAA-3´, 5´-CGGGCCTCTCGAACCAGTCC-3´, 5´-TTCAGCAGGACCACTTCCAT-3´) were used. For control cells, non-targeting (NT) gRNA was prepared by using negative control crRNA (NC1 from IDT: 5´-CGTTAATCGCGTATAATACG-3´) with tracrRNA. Two million freshly purified CD4^+^ CD25^−^ T cells were then transfected by nucleofection (Nucleofector 96-well Shuttle System, program EH100) with the RNP complexes and rested for 24 h in RPMI supplemented with 10% serum and further cultured under iTreg conditions for 72 h, as above.

### Flow cytometry

For surface staining, naïve CD4^+^ T cells or iTreg and control cells at 72 h of differentiation were washed with FACS buffer (2% FBS, 0.01% natriumazide in PBS) and stained with PE-conjugated CD45RO (BD Biosciences, Cat# 555493, RRID: AB_395884) and FITC-conjugated CD45RA (BD Biosciences Cat# 555488, RRID: AB_395879) or AF488-conjugated CD103 (BioLegend, Cat# 350208, RRID: AB_10641844) for 30 min at 4˚C. Intracellular staining of FOXP3 and HIC1 was performed using the eBioscience™ FOXP3/Transcription Factor Staining Buffer Set (Thermo Scientific, Cat# 00-5523-00) according to the manufacture’s protocol. Cells were stained with PE-conjugated FOXP3 (Thermo Fisher Scientific, clone PCH101, Cat# 12-4776-42, RRID: _AB151878; or clone 236 A/E7, Cat# 12-4777-41, RRID: AB_1944448) and AF647-conjugated HIC1 (Santa Cruz, Cat# sc-271499, RRID: AB_10650134) or corresponding isotype controls (Thermo Fisher Scientific Cat# 12-4321-42, RRID: AB_1518773 and BD Biosciences Cat# 557903, RRID: AB_396928). After staining, the cells were washed twice, resuspended in FACS buffer and acquired on BD LSRFortessa (BD Biosciences). The data were analyzed with FlowJo software (FlowJo LLC).

### Cell sorting

At 72 h of differentiation, CD103^+^ and CD103^−^ iTregs from four biological replicates were sorted using CD103 marker (AF488-conjugated CD103 (BioLegend, Cat# 350208, RRID: AB_10641844). Cell sorting was performed with the Sony SH800 Cell Sorter (Sony Biotechnology).

### Mass spectrometry

#### Sample preparation

Fluorescence-activated cell sorting (FACS)-based CD103^+^ and CD103^−^ sorted iTreg cells were lysed in a buffer containing 4% SDS in 50 mM TRIS-HCl (pH 8.0) and treated with Benzonase for 10 min at room temperature (RT). The lysate was clarified by centrifugation at 13,000 x g for 10 min, and protein concentration was determined using a DC Protein Assay (Bio-Rad). Twenty micrograms of protein was used for trypsin digestion. Dithiothreitol (10 mM) was added to the protein sample, followed by iodoacetamide to 20 mM, and incubated for 30 min in the dark. Proteins were digested using the Suspension Trapping (STrap) method as described [20]. Alkylated proteins were acidified with phosphoric acid to 1.2%, mixed with binding buffer (90% methanol, 100 mM TEAB, pH 7.55), and loaded onto STrap columns. After washing, proteins were digested overnight with MS-grade trypsin. The resulting peptides were eluted with 50 mM TRIS-HCl, 0.2% formic acid, and 50% acetonitrile, and dried using a SpeedVac.

#### Mass spectrometry analysis

Dried peptides were reconstituted in a formic acid/acetonitrile mixture, and 800 ng were analyzed using an EasynLC 1200 system coupled to an Orbitrap Fusion™ Lumos™ mass spectrometer (Thermo Scientific) in positive ion mode. Peptides were loaded onto a pre-column (20 × 0.1 mm) and separated over a 120-min gradient on a 75 μm x 15 cm analytical column (in-house packed with 3 μm Reprosil C18; Dr Maisch GmbH). The gradient elution profile for solvent B was as follows: 5–21% in 62 min, 21–36% in 48 min, 36–100% in 5 min, and held at 100% for 5 min, with a flow rate of 300 nL/min. The mobile phases were water with 0.1% formic acid (A) and 80% acetonitrile with 0.1% formic acid (B). Column temperature was maintained at 60 °C using a column oven.

#### FAIMS-DIA acquisition

Data Independent Analysis (DIA) was performed in positive mode with a mass resolution of 120,000, scan range (m/z) of 395–1005, normalized AGC target of 175%, and maximum injection time of 50 ms, using 30 optimized variable mass-windows. A high-field asymmetric ion mobility spectrometer (FAIMS) with compensation voltages of -50 V and − 70 V was used. The MS/MS scan range (m/z) was set to 180–2000, with loop control set to all. Normalized collision energy was 28%, Orbitrap resolution 30,000, maximum injection time 52 ms, and normalized AGC target 2000%.

#### Data processing

Raw files were converted to .htrms files using htrms converter and analyzed using Spectronaut (v18). Trypsin was selected as the digestion enzyme, with carbamidomethylation as a fixed modification, and methionine oxidation and N-terminal acetylation as variable modifications. False discovery rates (FDRs) were set at 0.01. Heatmap and volcano plot were generated with heatmapper [21], and GraphPad Prism8 software (GraphPad Software, Inc), respectively, using a corrected p value < 0.05 and fold-change (log2FC) > 0.58.

#### Functional data analysis

Functional enrichment analysis was performed using Ingenuity Pathway Analysis (Qiagen). IPA pathways with p value < 0.05 were considered significantly enriched. The activation z score was calculated to predict activation or inhibition of pathways. Pathways with |z score| > 2 were considered significant.

#### Network analysis

To represent the key networks of differentially abundant proteins, the list was filtered using the CRAPome database [22], removing common contaminants, such as keratins and cytoskeletal components. To visualize these and their associated protein-protein interaction (PPI) networks, Cytoscape (v3.10.2) [23] was used together with the STRING (version 12) database [24] and the ClueGO (version 2.5.10) plug-in [25]. The pathway enrichment analysis was made against the background of detected proteins for reference. For visualization, the singletons and lone networks with less than five members were removed.

### Western blotting

Cell samples were lysed in RIPA buffer (Thermo Fisher, Cat# 89901), supplemented with 1X Halt™ Protease and Phosphatase Inhibitor Cocktail (Thermo Fisher, Cat# 1861281). Cell lysates were sonicated (Bioruptor UCD-200; Diagenode) and cleared by centrifugation at 18,000 × g for 20 min. Protein concentration was determined using the DC Protein Assay (Bio-Rad). After boiling in 6 × loading dye (330 mM Tris-HCl, pH 6.8; 330 mM SDS; 6% β-ME; 170 µM bromophenol blue; 30% glycerol), samples were loaded on 4 - 20% gradient gel (MINI-PROTEAN TGX gel, Bio-Rad) and transferred to PVDF membranes (Trans-Blot Turbo Transfer Packs, BioRad). Membranes were blocked with 5% BSA-TBST (Tris-buffered saline and 0.1% Tween 20) and incubated with anti-human FOXP3 antibody (Thermo Fisher Scientific Cat# 14-4776-82, RRID: AB_467554), anti-human STAT4 antibody (Cell Signaling Technology Cat# 2653, RRID: AB_2255156), anti-human PRDM1 antibody (Cell Signaling Technology Cat# 9115, RRID: AB_2169699), and anti-human IKZF3 antibody (Abcam Cat# ab139408, RRID: N/A) overnight at 4 °C. After washing with TBST, membranes were incubated with HRP–conjugated secondary anti-rat IgG (Thermo Fisher Scientific Cat# A18733, RRID: AB_2535510) or HRP-conjugated anti-rabbit IgG (BD Biosciences Cat# 554021, RRID: AB395213) in 5% BSA-TBST at RT for 1 h. The housekeeping protein β-actin was used as a loading control (anti-β-actin primary antibody, Sigma-Aldrich Cat# A5441, RRID: AB_476744 and secondary antibody m-IgGκ BP conjugated to HRP, Santa Cruz Biotechnology Cat# sc-516102, RRID: AB_2687626).

### Suppression assay

The immunosuppressive capacity of iTreg or Th0 control cells was analyzed in a co-culture model with CD4^+^CD25^−^T cells (responder cells) as described previously [9]. Briefly, responder cells were isolated from peripheral blood buffy coats, as described under CD4^+^CD25^−^ T cell isolation and differentiation section, and stained with 5 µM of CellTrace™ Violet (Life Technologies, # C34557). Labeled responder cells were then co-cultured with iTreg or Th0 cells, and CD103^+^ or CD103^−^ Tregs at different ratios (responder cell: iTreg/Th0, and responder cell: CD103^+^/CD103^−^ Treg ratio 1:1, 1:0.5, 1:0.25) in X-vivo 15 medium and in the presence of plate-coated anti-CD3 (150 ng/well) and soluble anti-CD28 (125 ng) for four days. As controls, responder cells were cultured alone either under same conditions as the Th0/iTreg co-cultures (positive control) or left unstimulated (negative control). On day four, data were acquired on BD LSRFortessa flow cytometer (BD Bioscience) and analyzed with FlowJo software (FlowJo LLC).

### Mass cytometry

#### Antibody labelling and cell staining

A 25-parameter mass cytometry antibody panel was designed to study the expression of Treg markers during early cell differentiation. Maxpar antibodies used in this study were obtained from Standard BioTools (Table 1). Antibodies that required in-house conjugation were labeled with either cadmium metals, using the Maxpar MCP9 Antibody Labeling Kit (Standard BioTools), or lanthanide metals, using the Maxpar X8 Antibody Labeling Kit (Standard BioTools), according to manufacturer’s instructions (Table 1 highlighted in bold). To determine specificity and optimal concentration, we performed validation and titration for all conjugated antibodies. Further, to ensure minimal technical variation, we prepared a master mix of all conjugated antibodies, aliquoted and stored at -80 °C.

**Table 1:** Metal-conjugated antibodies specific to Treg-associated markers

Conjugate	Antibody	Clone	Company	Catalog
111Cd	CD278 (ICOS)	C398.4 A	BioLegend	313,502, RRID: AB_416326
113Cd	CD15s	CSLEX1	BD Biosciences	551,344, RRID: AB_394156
116Cd	CD39	A1	BioLegend	328,221, RRID: AB_2563747
142Nd	CD152 (CTLA4)	14D3	Thermo Fisher Scientific	14-1529-82, RRID: AB_467512
143Nd	HLA-DR	L243	St. BioTools	3143013B
145Nd	CD45RO	UCHL1	BioLegend	304,239, RRID: AB_2563752
149Sm	CD25 (IL2Rα)	2A3	St. BioTools	3149010B
150Nd	CD134 (TNFRSF4)	ACT35	St. BioTools	3150023B
151Eu	CD103 (ITGAE)	Ber-ACT8	St. BioTools	3151011B
154Sm	CD120b (TNFR2)	3G7A02	BioLegend	358,402, RRID: AB_2562150
155Gd	CD27	L128	St. BioTools	3155001B
158Gd	CD194 (CCR4)	L291H4	St. BioTools	3,158,032 A
159 Tb	TIGIT	MBSA43	St. BioTools	3159038B
160Gd	CD28	CD28.2	St. BioTools	3160003B
162Dy	FOXP3	PCH101	St. BioTools	3,162,011 A
147Sm	FOXP3	237/E7	Thermo Fisher Scientific	14-4777-82, RRID: AB_467556
163Dy	CD183 (CXCR3)	G025H7	St. BioTools	3163004B
165Ho	CD223 (LAG3)	11C3C65	St. BioTools	3165037B
167Er	CD197 (CCR7)	G043H7	St. BioTools	3,167,009 A
168Er	CD73	AD2	BioLegend	344,002, RRID: AB_2154067
169Tm	CD366 (TIM3)	F38-2E2	St. BioTools	3169028B
172Yb	CD38	HIT2	St. BioTools	3172007B
173Yb	CD184 (CXCR4)	12G5	St. BioTools	3173001B
175Lu	CD279 (PD1)	EH12.2H7	St. BioTools	3175008B
176Yb	CD127 (IL-7Rα)	A019D5	St. BioTools	3176004B
209Bi	CD137 (TNFRSF9)	4B4-1	St. BioTools	3209015B

Cells were stained with 2.5 µM Cell-ID Cisplatin (Standard BioTools, Cat# 201064) for dead cell discrimination. Surface and intracellular antibody staining was performed according to Maxpar Nuclear Antigen Staining with Fresh Fix protocol (Standard BioTools). Briefly, cells were first stained with a cocktail of surface antibodies (Table 1), followed by intracellular staining of FOXP3. Cells were finally fixed with 1.6% formaldehyde solution (Thermo Fisher, Cat# PI28906) and incubated with Cell-ID intercalator solution at concentration of 85 nM (Standard BioTools, Cat# 201192 A) overnight at 4 ºC. Samples were then frozen at -80 ºC until analysis.

Before sample acquisition, cells were thawed and washed once with Maxpar cell-staining buffer (Standard BioTools, Cat# 201068) and twice with Cell Acquisition Solution (Standard BioTools, Cat# 201248). Cells were then filtered through a 35-µm filter cap FACS tubes (BD Biosciences) and diluted to 0.5 × 10^6^ cell/ml with the Cell Acquisition Solution (Standard BioTools) containing 10% EQ Four Element Calibration Beads (Standard BioTools). Data were acquired on Helios mass cytometer (Standard BioTools), for all samples, 100,000 events were collected.

#### Data analysis

Data analysis was performed using R version 4.3.0. Data were pre-processed using the flowCore (v. 2.12.2) [26] package and transformed using hyperbolic arcsine transformation with the cofactor of 5. The cells were clustered using the FlowSOM (v. 2.8.0) package [27], followed by metaclustering with the ConsensusClusterPlus (v. 1.64.0) package [28]. A combination of unsupervised (overclustering) and supervised clustering (removal and merging) based on known cell-specific markers was performed. Heatmaps were clustered using hierarchical clustering with Euclidean distance as the dissimilarity metric and average linkage as the agglomeration method. For the Th0/iTreg (72 h) dataset, we initially used 12 clusters and then removed clusters representing either debris (no expression of lineage markers) or doublets (two or more lineage-specific markers). The remaining clusters were then merged based on lineage marker expression, resulting in a total of six clusters for the 72 h (Th0 and iTreg) data.

To statistically test the changes in cell type proportions, we used linear mixed effects modeling (LME) implemented in the lmerTest (v. 3.1-3) package. For the NT/FOXP3 CRISPR and Th0/iTreg 72 h data, we used the following formula for each functional mass cytometry marker: *expression ~ celltype + (1|pair/sample)*, where the cell-type is treated as a fixed effect and the pairing and sample as a nested random effect. The resulting p-values were FDR-corrected using the Benjamini-Hochberg method.

### Quantification and statistical analyses

Boxplots, bar plots and line plots were done with GraphPad Prism8 software (GraphPad Software, Inc.). At least three independent biological replicates were performed for each experiment unless otherwise stated in the figure legend. The number of biological replicates and the related statistical methods are described within figure legends. Statistical significance was concluded when *p* < 0.05.

## Results

### In vitro-induced FOXP3^+^ Treg cells are suppressive

**Fig. 1:**
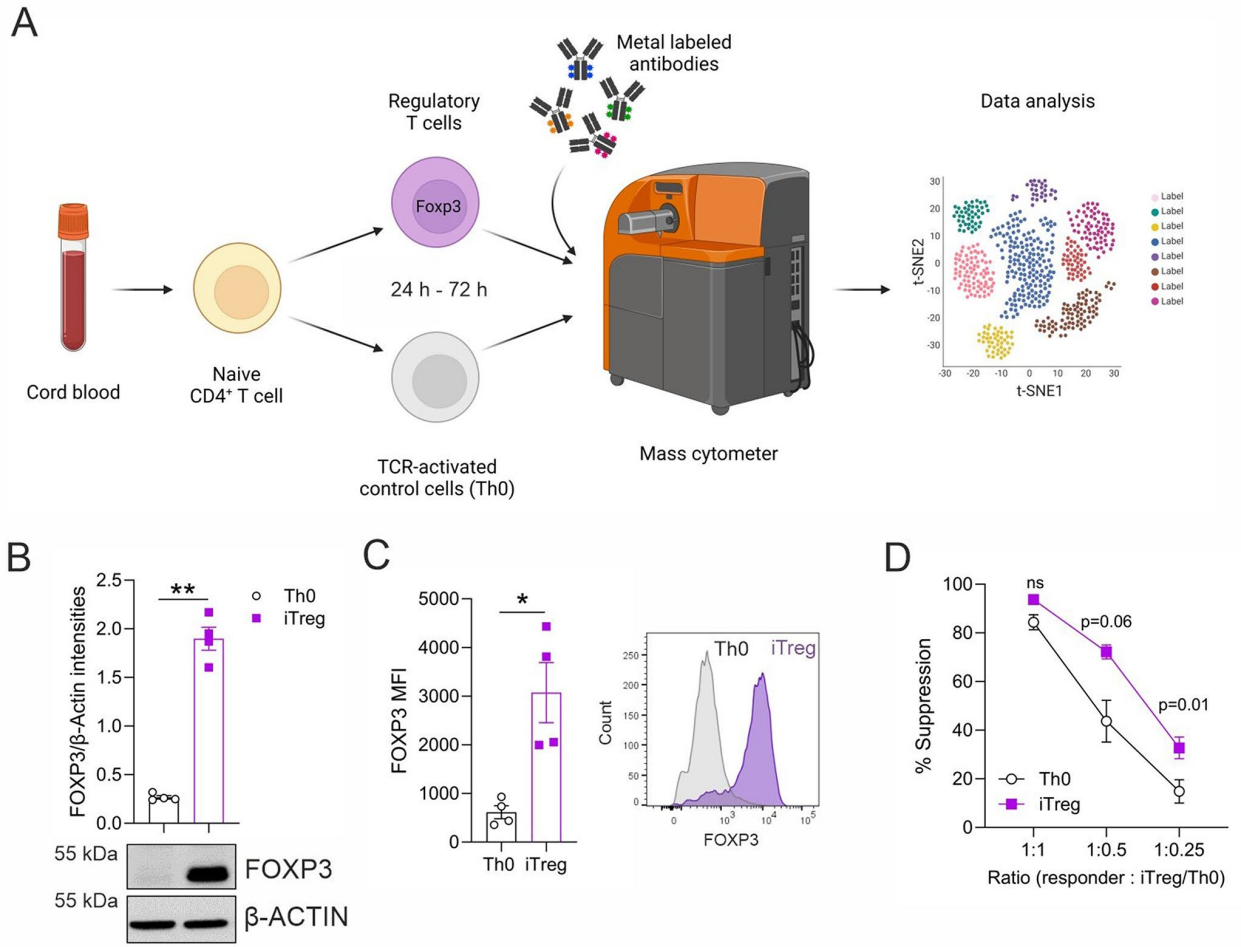
In vitro-induced Tregs express FOXP3 and are suppressive. (**A**) Workflow. Naïve CD4^+^ CD25^−^ T cells from four individual umbilical cord blood donors were cultured under iTreg polarizing conditions for 24 and 72 h and were analyzed by mass cytometry. CD4^+^ T cells activated by anti-CD3 and anti-CD28 were used as controls (Th0). A 25-parameter mass cytometry panel was designed to study the expression of Treg markers during early cell differentiation. (**B-C**) Expression of FOXP3 protein in iTreg at 72 h of differentiation was evaluated by western blot, normalized to β-actin (**B**) and intracellular staining using flow cytometry (FOXP3 Ab clone PCH101), showing mean fluorescent intensities (MFI) (**C**). (**D**) The suppressive capacity of these iTregs was evaluated by co-culturing them with fluorescence-labeled CD4^+^CD25^-^ responder T cells at different ratios using flow cytometry. The percentage of suppression is shown. Plots in (**B**-**D**) show mean ± SEM from four individual biological replicates. Statistical significance is calculated using paired T-tests (ns, not significant; * *p* < 0.05, ** *p* < 0.01)

To characterize human iTregs by mass cytometry, CD4^+^CD25^−^ T cells were isolated from four individual umbilical cord blood donors and differentiated into iTreg and TCR activated control cells (Th0) (Fig. 1A). At 72 h of differentiation, western blot analysis showed that FOXP3 protein was significantly upregulated in iTregs compared to control cells (Fig. 1B). Further, flow cytometry-based intracellular staining revealed that 85–90% of the iTregs were FOXP3^+^ (Fig. 1C). Moreover, iTregs suppressed the proliferation of fluorescence-labeled CD4^+^CD25^−^ T cells to a greater extent than the control in a co-culture system (Fig. 1D), indicating that they were functional.

### Multi-marker profiling of human iTreg and Th0 control cells using mass cytometry

**Fig. 2:**
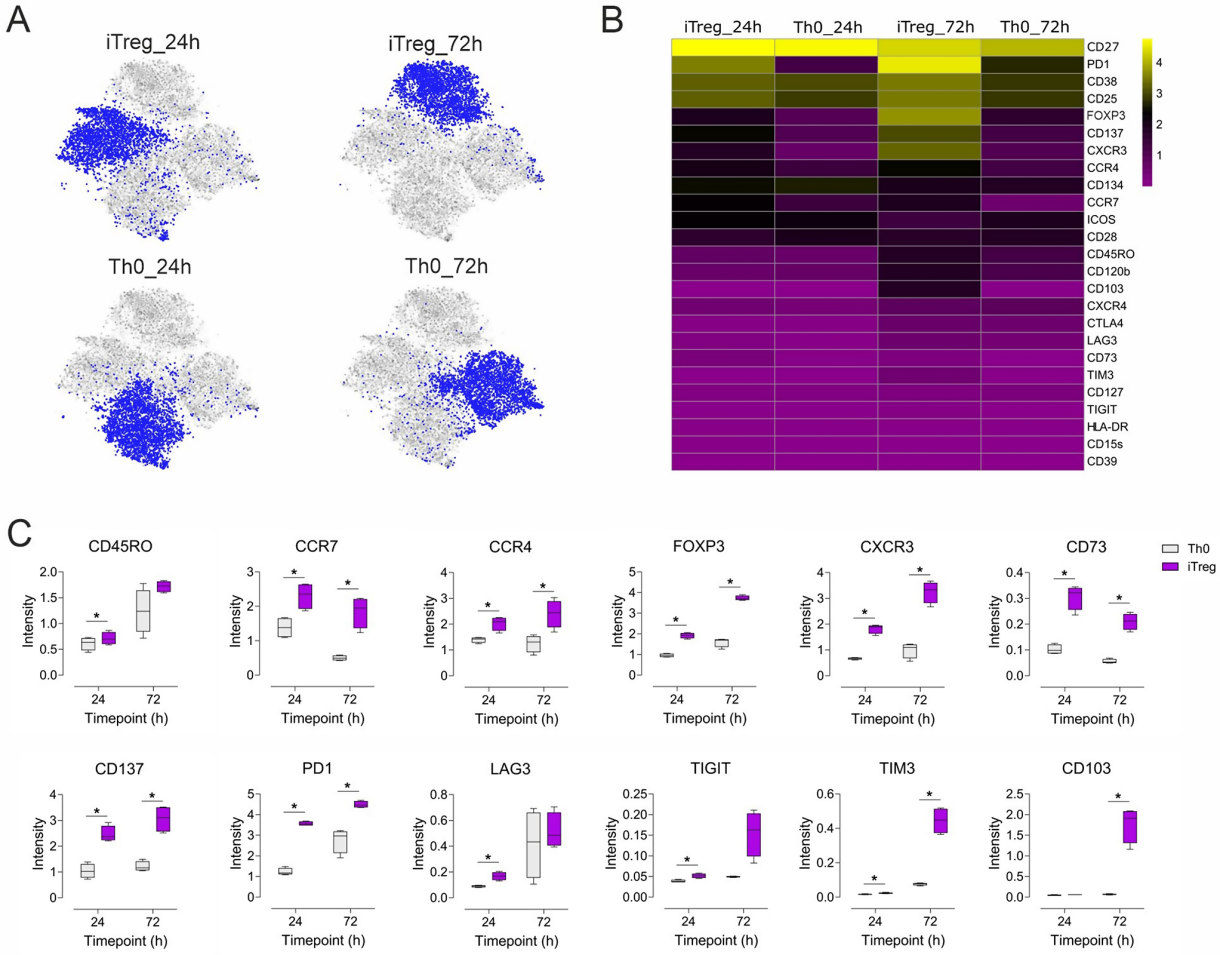
Multi-marker profiling of human iTreg and Th0 control cells using mass cytometry. (**A**) t-SNE visualization of marker intensity distribution from all four individuals combined are shown for iTregs and control cells activated by anti-CD3 and anti-CD28 (Th0) at 24 and 72 h of differentiation. (**B**) The median intensities of the 25 markers at 24 and 72 h of iTregs and Th0 cells for four individual biological replicates are shown as a heatmap. (**C**) Selected differentially expressed markers are shown as box plots. Boxplot represents median and interquartile range, and whiskers extend to maximum and minimum values. Data are shown for four individual biological replicates. Statistical significance is calculated using paired T-test (* FDR < 0.05)

A panel of 25 antibodies, specific to a range of markers associated with human Tregs, and high-resolution mass cytometry were used to profile human iTregs during early differentiation. Marker intensity distribution of all combined markers from the four individuals showed clear and distinct clusters for iTreg and Th0 cells at 24 and 72 h of differentiation (Fig. 2A). The median expression of the 25 selected markers in iTreg and Th0 cells at each time point are summarized as a heatmap (Fig. 2B). FOXP3 and IL2 receptor α chain (CD25), widely used to determine Treg lineage [8, 29, 30], were expressed in iTregs accompanied by the lack of IL7 receptor (CD127) (Fig. 2B). Expression of the co-inhibitory receptor PD1 was also strongly induced in iTregs. Moreover, the co-stimulatory receptors ICOS, CD134, CD27 and CD28 [31] were expressed in Th0 and iTreg cells. Of these, CD27 was the top upregulated marker in both cell types, confirming its constitutive expression on CD4^+^ T cells, including CD4^+^FOXP3^+^ Tregs [32]. In contrast, other co-inhibitory molecules (TIGIT, CTLA4, TIM3, and LAG3) were expressed at a low level. CD39 and CD73 (Fig. 2B), two molecules associated with adenosine mediated suppression [33], were not detected in iTregs.

Differential expression analysis further revealed that 18 out of the 25 markers were significantly induced in iTregs in at least one time point (Fig. 2C, S2). Among them, CCR4, CCR7, CXCR3, CD73, PD1, CD137, and FOXP3 were upregulated to a greater extent in iTregs compared to controls. Expression levels of the co-inhibitory markers TIGIT, TIM3 and LAG3 were low but significantly higher during early iTreg differentiation (24 h) and further increased with time (72 h). Interestingly, levels of CD103, also known as the alpha chain of the integrin αEβ7, were strongly and selectively expressed at 72 h in iTregs (Fig. 2C).

### Immunophenotypic characterization of the human iTreg compartment reveals a CD103^+^ subpopulation

**Fig. 3:**
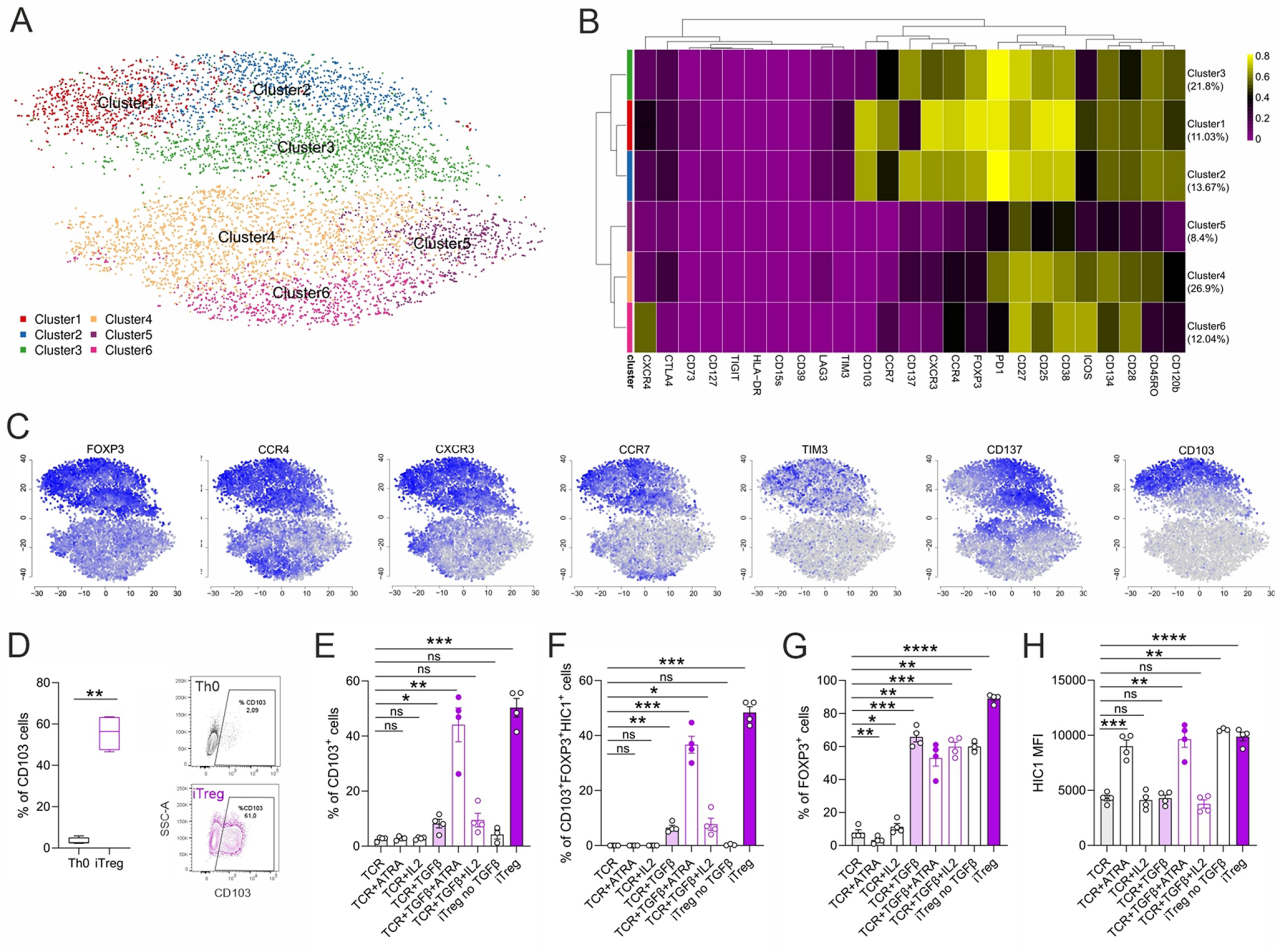
Immunophenotypic characterization of the human iTreg compartment reveals a CD103^+^ subpopulation. (**A**-**C**) A panel of 25 Treg-associated markers was utilized to characterize human iTreg and control cells activated by anti-CD3 and anti-CD28 (Th0) at 72 h of differentiation by mass cytometry. (**A**) tSNE plot of iTreg and Th0 cells, from all four individuals combined, is shown. (**B**) A heatmap of the normalized marker expression in each cluster is shown. (**C**) The expression profiles of selected markers are displayed as tSNE plots. (**D**) The percentage of CD103^+^ cells was validated at 72 h in Th0 and iTreg cells by flow cytometry for five biological replicates. Boxplots represent median and interquartile range, and whiskers extend to maximum and minimum values. Representative histograms are shown on right panel. (**E**-**H**) The percentage of single CD103^+^ (**E**), triple CD103^+^FOXP3^+^HIC1^+^ (**F**), single FOXP3^+^ cells (**G**), and the mean fluorescence intensities (MFI) of HIC1 intracellular expression (**H**) determined by flow cytometry, are shown from naïve CD4^+^ T cells cultured for 72 h under Th0 condition, iTreg differentiation condition, or Th0 in the presence of IL2, ATRA and TGFβ alone or in combinations. Plots in **E**-**H** show mean ± SEM of four biological replicates. Statistical significance in **D**-**H** is calculated using paired T-tests (∗ *p* < 0.05, ∗∗ *p* < 0.01, ∗∗∗ *p* < 0.001, ∗∗∗∗ *p* < 0.0001)

Next, unsupervised clustering analysis was applied to evaluate the 25-marker composition and expression profile of the iTreg and Th0 compartment at 72 h of differentiation. t-SNE visualization revealed six clusters (Fig. 3A). The median expression of every marker for each cluster is displayed as a heatmap (Fig. 3B). iTregs were clearly separated from control cells and were represented in three clusters (1, 2 and 3) (Fig. 3A, B). FOXP3, along with CD137, CD103, CCR4, CCR7, CXCR3, CD73, TIGIT and TIM3, were predominantly expressed in the iTreg compartment (Fig. 3B, C, S3A). Additionally, iTregs strongly upregulated the expression of PD1, CD38, and CD27, which were also induced upon TCR activation, as represented in the Th0 compartment (clusters 4 and 6) (Fig. 3B, S3A). Cluster 5 within the Th0 compartment showed reduced expression of all markers. Interestingly, CD103^+^ cells were exclusively detected in the FOXP3^+^ iTreg population, represented by two clusters (1 and 2, totaling 24.7%), forming almost half of the Treg compartment. This indicates that signals other than T cell activation are needed to induce CD103 expression (Fig. 3B, C). Cluster 1 revealed the highest expression of Treg-associated molecules, except CD137, which was expressed at low levels. Moreover, CD137^+^ cells were represented in two clusters (2 and 3, totaling 34%) and of these, cluster 3 (20.4%) was CD103 negative (Fig. 3B, C). Overall, these results indicate that iTregs are divided into CD103^+^FOXP3^+^ and CD103^−^FOXP3^+^ populations, which were further confirmed by flow cytometry (Fig. 3D).

CD103 is a surface molecule that marks both intestinal lymphocytes and a particularly suppressive subtype of Tregs [12]. Several studies have shown that TGFβ1 induces the expression of CD103 in human Tregs [14, 34, 35]. To confirm these findings, we stimulated TCR-activated T cells (Th0) in presence of either TGFβ, ATRA and IL2 alone or combinations of ATRA or IL2 with TGFβ, and measured the expression of CD103 by flow cytometry. In agreement with previous reports [34, 36], our data revealed that TGFβ alone or combined with IL2 induced the expression of CD103. However, the combination of TGFβ and ATRA, in the absence of IL2, strongly enhanced CD103 expression to levels comparable to those detected on iTregs. IL2 or ATRA alone did not upregulate CD103 (Fig. 3E, S3B). Moreover, the CD103^+^ iTregs co-expressed the TF FOXP3 and the ATRA-inducible TF HIC1 at 72 h of differentiation (Fig. 3F). Interestingly, although TCR + TGFβ induced about 60% of FOXP3^+^ cells, the percentage of cells expressing CD103 remained low (Fig. 3G, S3C). Similar results were observed in TCR- and ATRA-treated naïve CD4 + T cells with enhanced levels of HIC1 (Fig. 3H), indicating that CD103 expression is controlled by complex regulation network.

### FOXP3 negatively regulates CD103 expression

**Fig. 4:**
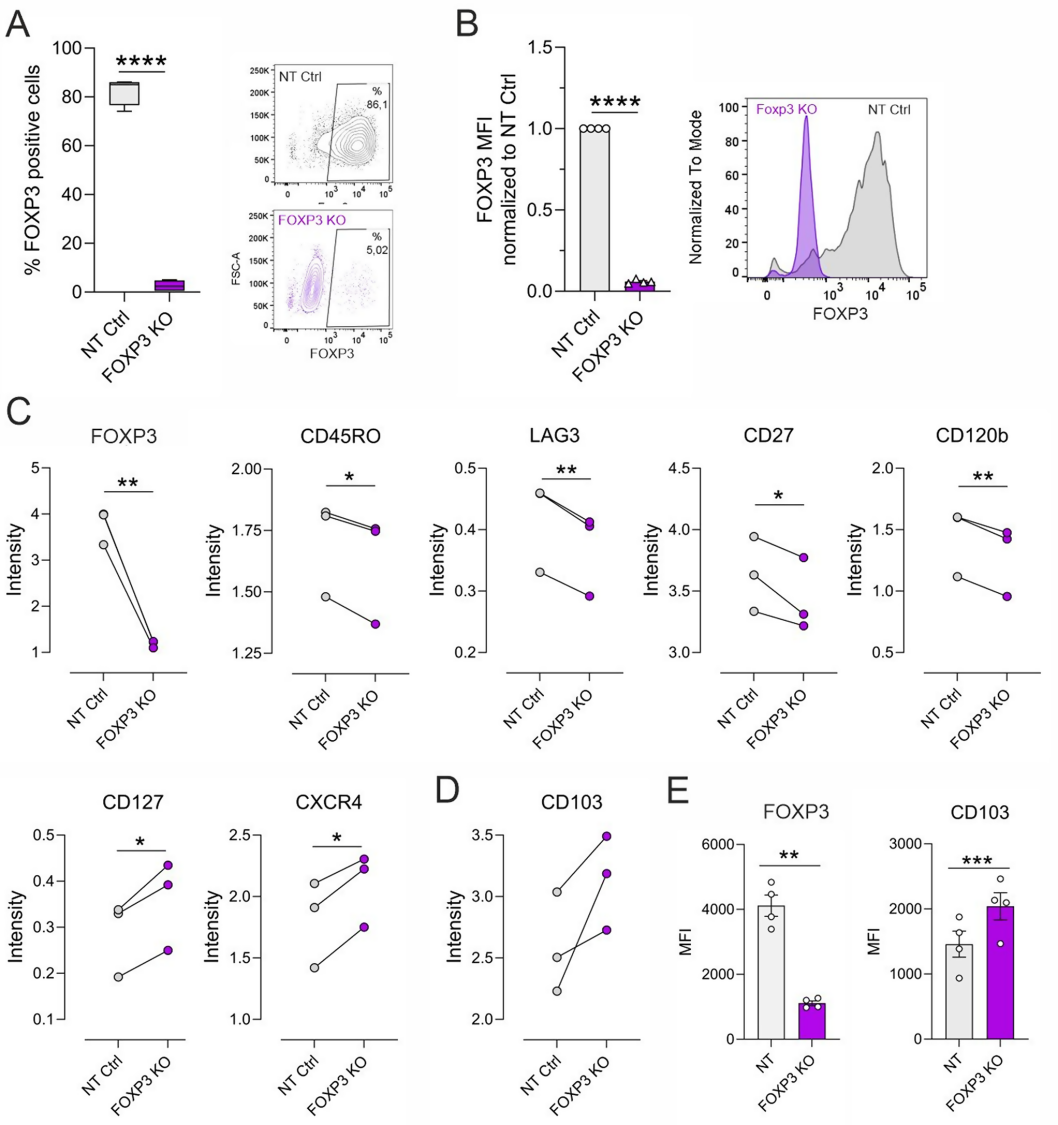
Effect of FOXP3 silencing on iTreg associated surface markers. (**A**-**B**) Ablation of FOXP3 using CRISPR-Cas9 was confirmed by flow cytometry at 72 h of iTreg differentiation. Percentage of FOXP3^+^ cells (**A**) and mean fluorescence intensities (MFI) of FOXP3 relative to NT Ctrl (FOXP3 Ab clone 237/E7) (**B**) are shown as bar plots for four individual donors (left). Representative dot plots and histograms, respectively, are shown (right). (**C**) Based on mass cytometry analysis, the intensity of differentially expressed markers in NT Ctrl and FOXP3 KO iTregs are displayed as line plots for three individual donors. Statistical significance is assessed using linear mixed effects modeling, as described in the Method section. (**D)** CD103 intensity, assessed by mass cytometry in NT Ctrl and FOXP3 KO iTregs at 72 h is shown. (**E**) CD103 upregulation in FOXP3 KO iTregs was further validated by flow cytometry, showing MFI of FOXP3 (Ab clone 237/E7) and CD103 in four biological replicates. Plots in (**A**,**B** and **E**) show mean ± SEM. Statistical significance in **A**,**B**,**E** is calculated using paired T-tests (∗ *p* < 0.05, ∗∗ *p* < 0.01, ∗∗∗ *p* < 0.001, ∗∗∗∗ *p* < 0.0001)

To determine the role of FOXP3 during early human iTreg differentiation and its effect on CD103, we ablated FOXP3 in iTregs using the CRISPR/Cas9 system and studied its effect on the expression of the 25 Treg-associated markers with mass cytometry. Ablation of FOXP3 protein was confirmed by intracellular staining using flow cytometry at 72 h of iTreg differentiation (Fig. 4A, B). Based on marker intensity distribution using mass cytometry, we noted that the NT control cells clustered differently than the FOXP3 knockout (KO) iTregs, despite the inter-donor variability (Fig. S4A). Besides FOXP3, CD45RO, CD120b, CD27 and LAG3 were also significantly downregulated upon FOXP3 ablation. CXCR4 and CD127 were upregulated in FOXP3-deficient iTregs, suggesting they are negatively regulated by FOXP3 (Fig. 4C). Further, while FOXP3 silencing modestly downregulated TIM3, TIGIT and PD1, it induced expression levels of CD38, CD25, CD103 and CD73, albeit not statistically significantly, underscoring inter-donor variations (Fig. S4B). Interestingly, CD103 levels were consistently greater in FOXP3 KO Tregs compared to control (Fig. 4D), and this was further validated by flow cytometry (Fig. 4E), indicating that FOXP3 negatively regulates CD103 expression.

### CD103^+^ iTregs have a more immunosuppressive activity than their CD103^−^ counterparts

**Fig. 5:**
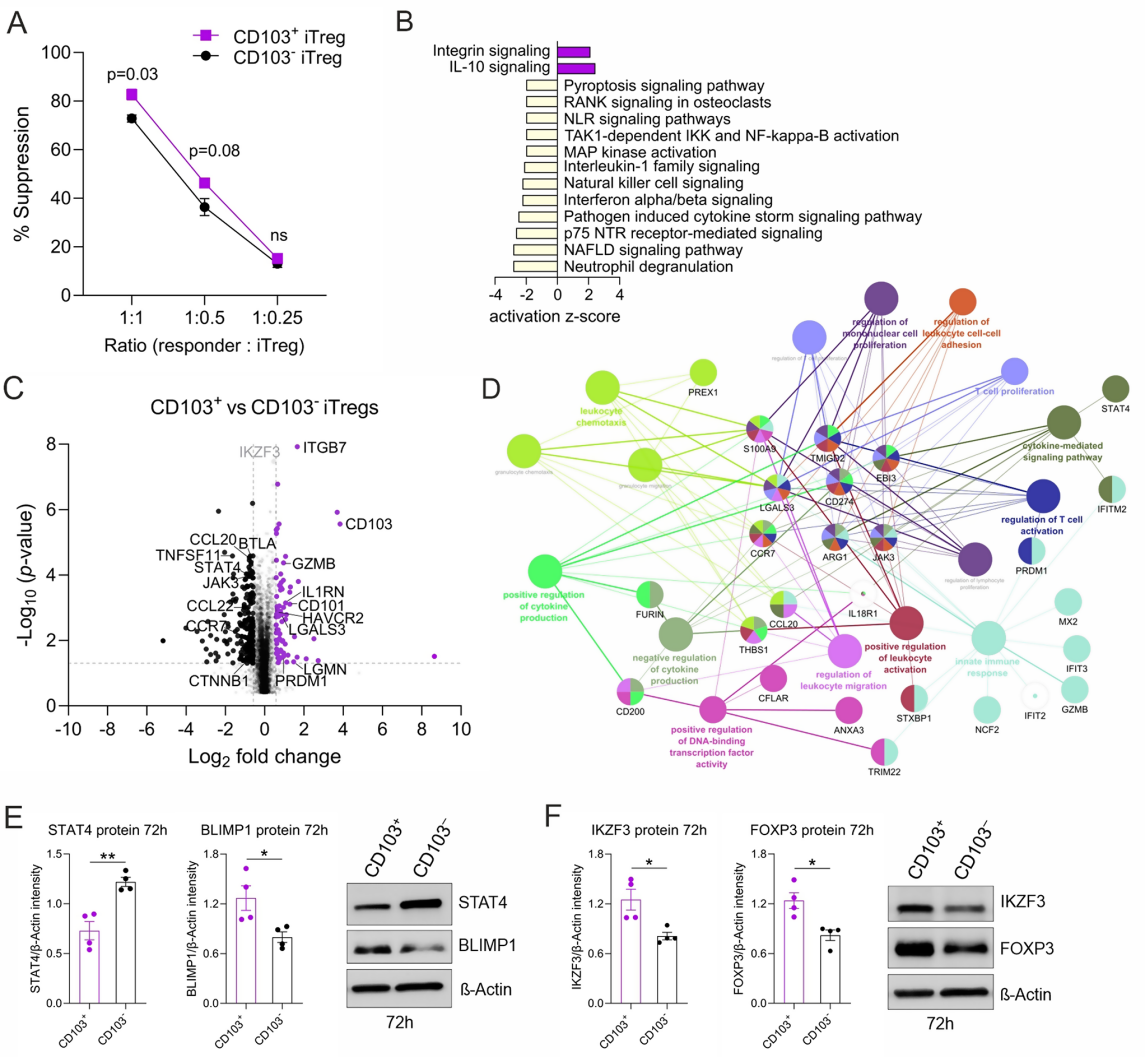
CD103^+^ iTregs co-express several immunosuppressive factors. (**A**) At 72 h of iTregs differentiation, CD103^+^ and CD103^−^ iTregs were sorted and co-cultured with fluorescence-labeled CD4^+^CD25^−^ responder T cells at different ratios to study their suppressive capacity by flow cytometry. The percentage of suppression is shown. (**B**) Mass spectrometry-based proteomics was performed on sorted CD103^+^ and CD103^−^ iTregs for four biological replicates. IPA was used to identify signaling pathways that are significantly altered between CD103^+^ and CD103^−^ iTregs. Pathways with a p value < 0.05 and |Z score| > 2 were considered to be significantly enriched. The activation Z score was calculated to predict activation or inhibition of pathways. (**C**) Volcano plot highlights the Treg-associated proteins that are differentially expressed between sorted CD103^+^ and CD103^−^ Tregs at 72 h of polarization with corrected p value of < 0.05 and log2FC > 0.58. Upregulated proteins are in purple, and downregulated proteins are in black. (**D**) Key networks of differentially abundant proteins and their associated protein-protein interaction (PPI) networks were visualized using Cytoscape together with the STRING database. The pathway enrichment analysis was made against the background of detected proteins for reference. (**E**-**F**) Expression of STAT4, and BLIMP1 (**E**, left and middle panels), and FOXP3 and IKZF3 protein levels (**F**, left and middle panels) in CD103^+^ and CD103^−^ iTreg at 72 h of differentiation were evaluated by western blot. Representative immunoblots are shown (**E**, right panel and **F**, right panel). Plots in (**A**,**E**,** F**) show mean±SEM from four individual biological replicates. Statistical significance is calculated using paired T-tests (ns, not significant; * *p* < 0.05, ** *p* < 0.01)

CD103^+^ Tregs have a stronger suppressive function than CD103^−^ Tregs in mice [37, 38]. To further characterize human CD103^+^ iTregs, we sorted the CD103^+^ and CD103^−^ iTreg populations (CD103^+^FOXP3^+^ and CD103^−^FOXP3^+^) and examined their suppressive properties in vitro. CD103^+^ iTregs had a modest but slightly higher suppressive activity than CD103^−^ iTregs (Fig. 5A).

Next, we performed mass-spectrometry-based proteomics to gain insights into the biological function of immunosuppressive CD103^+^ iTregs and their CD103^−^ counterparts. Principal component analysis (PCA) revealed that CD103^+^ iTregs have a different protein profile than CD103^−^ iTregs (Fig. S5A). We further detected 289 differentially expressed proteins between the two populations (corrected p value < 0.05, log2FC > 0.58) (Table S1). Among these, 77 proteins were upregulated while 212 proteins were downregulated in CD103^+^ iTregs, when compared to the CD103^−^ fraction. The top 80 differentially expressed proteins are shown as a heatmap (Fig. S5B). Ingenuity Pathway Analysis showed that proteins associated with IL10 and integrin signaling were significantly enriched in the CD103^+^ iTreg population, but inflammatory response pathways (MAP kinase and NFκB activation, cytokine storm, IFNα/β, IL1 family and NLR signaling pathways) were suppressed, indicating an anti-inflammatory phenotype (Fig. 5B).

ITGB7, an exclusive dimerization partner of CD103 [39], was among the top upregulated proteins in the CD103^+^ iTreg population (Fig. 5C). Levels of CD103 were found to positively correlate with the expression of cysteine-endoprotease legumain (LGMN) and Galectin-3 (LGALS3), both of which are induced in human Tregs [40] and enhance FOXP3 expression [41]. Furthermore, several proteins that promote Treg function in mice and human (IL1RN, CD101, TIM3 (HAVCR2), PRDM1 (BLIMP1), and GZMB) were significantly enriched in the CD103^+^ iTreg population (Fig. 5C). TIM3^+^ Tregs negatively regulate proinflammatory immune responses [42, 43] with superior suppressive function in head and neck cancer patients [44]. An immunomodulatory role of the surface molecule CD101 and the naturally occurring IL1 inhibitor (IL1RN) in Tregs have also been found in murine disease models [45, 46]. Interestingly, by lowering the fold change (FC) threshold (log2FC to 0.46), IKZF3 was among the top upregulated targets in CD103^+^ iTregs. IKZF3 is part of the FOXP3, RUNX1 [47] and HIC1 complex [48] that promotes the differentiation of functional Tregs. Upregulation of BLIMP1, IKZF3 and FOXP3 protein in CD103^+^ iTregs was further confirmed by western blotting (Fig. 5E, F).

Additionally, CD103^+^ iTregs exhibit lower expression levels of proteins known to negatively impact immunosuppression (e.g., STAT4 [49], and CTNNB1 (β-catenin) [50], as well as the inhibitor receptor BTLA, a member of the CD28 immunoglobulin superfamily [51]. Lower levels of STAT4 protein in CD103^+^ iTregs than CD103^−^ iTregs were further validated by western blotting (Fig. 5E). Notably, CD103 levels negatively correlated with proinflammatory molecules (CCL22, CCL20, TNFSF11 (RANKL), JAK3, and NLRP3 [52]) (Fig. 5C). Network analysis of the most abundant differently expressed proteins revealed clusters relevant for T cell activation, proliferation and cytokine mediated signaling (Fig. 5D). These findings indicate that the CD103^+^ iTregs possess an immunosuppressive phenotype.

## Discussion

Tregs represent a unique subpopulation of T lymphocytes that maintain immune equilibrium. They can be phenotypically and functionally heterogeneous and are composed of effector/memory Tregs and naïve Tregs (3–7).

Here, we used high-dimensional single-cell mass cytometry, together with a panel of 25 Treg-associated markers, to characterize human iTregs during their early stages of differentiation. Our data revealed that iTregs at 72 h of differentiation showed a CD25^+^FOXP3^+^CCR7^+^CD127^−^ phenotype with immunosuppressive properties. Moreover, these cells further expressed the memory T cell marker CD45RO and the co-stimulatory molecule ICOS [53], CD134 [54], CD28 and CD27 [55]. Loss of CD27 has been previously reported on fully differentiated effector T cells, but it is retained on central memory T cell phenotypes [32], indicating that iTregs in the present study share common features with memory Tregs.

Tregs suppress a variety of physiological and pathological immune responses via either direct or indirect mechanisms [56]. The inhibitory activity of Tregs, through cell–cell contact with immune cells occurs mainly via the co-inhibitory receptors, including CTLA4 [57] and PD1 [58], which have been targeted clinically to improve anti-tumor T-cell responses [10]. In the present study, PD1 was strongly upregulated, but other co-inhibitory molecules, including CTLA4, LAG3, TIGIT and TIM3, were weakly expressed during early human iTregs differentiation. TIGIT, LAG3 and TIM3 were transiently upregulated on a small fraction of CD4^+^FOXP3^+^ Tregs in the normal circulation [59]. These co-inhibitory receptors had distinct and specific roles in regulating immune responses, particularly at sites of tissue inflammation [59], which would explain their rather low abundance on iTregs at early stages of differentiation. We further showed the upregulation of the costimulatory molecule CD137 (TNFRSF9) on a fraction of iTregs. Interestingly, Tregs expressing CD137 have a stronger suppressive capacity than the CD137 negative counterpart [60–62]. Still, more studies are needed to reveal the precise role of CD137 in Treg expansion and suppressive function, particularly in the human system.

Additionally, iTregs expressed molecules involved in the function of other Th cell fates, including the chemokine receptors CCR4 and CXCR3. These two have been identified in suppressive memory- and/or effector-like Treg subsets that allow them to migrate to peripheral tissues and accumulate in tumors [63–66]. Upregulation of CXCR3 is controlled by the Th1-related TF TBET, and enables the recruitment of immunosuppressive CXCR3^+^ Tregs to sites of Th1 cell-mediated inflammation [67–69]. CCR4 expression has been associated with a Th2-like Treg phenotype in organ tissues [70, 71]. Our findings suggest that iTregs have the potential to be recruited to tissues to regulate inflammatory processes.

The alpha chain of the integrin αEβ7 (ITGAE), known as CD103, is a key phenotypic marker of resident memory T cells in a variety of tissues, including tumors, regulating immune responses. However, most studies of CD103^+^ Treg function were done in mouse models. Human CD103^+^ tissue-resident memory T cells are concentrated in lymphoid and mucosal sites, including the skin and small intestine [72, 73]. Recently, CD4^+^CD103^+^ cutaneous resident memory T cells were also identified in the blood of healthy individuals, challenging the current understanding of the compartmentalization of CD4^+^ memory T cells within tissues [74]. However, little is known about the role of CD103 in human iTregs.

We found CD103 was upregulated on a subset of FOXP3^+^ iTregs during early stages of differentiation. Notably, in response to allogeneic dendritic cells, cord blood derived T cells expressed a much greater level of CD103 on suppressor cells than their adult counterparts [14], indicating that the naive subset of T cells is more likely to acquire CD103 upon TCR and cytokine signaling. Accumulating studies have further shown that TGFβ alone or in combination with IL2 upregulate the expression of CD103 [14, 34, 36]. In Tregs, CD103 is either directly responsive to FOXP3 or induced by TGFβ in a FOXP3-independent manner [12], although the role of TGFβ in the induction of CD103 remains controversial [14, 36]. We showed that TCR activation in combination with TGFβ and ATRA induced much higher levels of CD103 than TCR + TGFβ or TCR + TGFβ + IL2 activation. Moreover, CD103^+^FOXP3^+^ iTregs co-expressed the transcriptional regulator HIC1. HIC1 is an ATRA responsive gene in intestinal T helper cells and required for the optimal expression of CD103 in mice, facilitating the retention of T cells in the intestinal microenvironment [75]. Previously, we reported that the TF HIC1 contributes to suppressive function of human iTregs independently of FOXP3 [9]. Interestingly, FOXP3 suppresses expression of CD103 in iTregs, suggesting that CD103 might be under multifactorial and complex regulation. Moreover, this reciprocal inverse regulatory relationship between FOXP3 and CD103 during iTreg differentiation, could suggest a shift in cellular identity, potentially towards a TRM-like phenotype. However, a more comprehensive characterization of FOXP3-ablated iTregs is needed to speculate on their cellular identity and function. Additionally, TRM cell development, especially in humans, remains an area of active research and is not yet fully understood.

Furthermore, we found human CD103^+^ iTregs to be more suppressive than their CD103^−^ counterparts. Our proteomics results indicated a positive correlation between CD103 protein levels and pro-immunosuppressive molecules (CD101, BLIMP1, TIM3, IKZF3, and GZMB), while negative regulators of immunosuppression (BTLA, STAT4, and β-catenin) were impaired in CD103^+^ iTregs. Interestingly, both BLIMP1- and TIM3-expressing Tregs have been associated with high expression of GZMB, resulting in enhanced effector-like function with increased suppressive activity [43, 76, 77]. However, the TF BLIMP1 seems to prevent a full switch of Treg cells to an effector phenotype by reducing IL17 expression [78]. CD101, which is highly expressed on activated mucosal tissue-resident memory T cells [72], has also been suggested to regulate the balance between anti-inflammatory Treg cells and proinflammatory Th17 cells in mucosal tissue [79], and thus, CD103^+^ iTregs may be associated with Treg identity and functionality. Further studies are necessary to better understand the mechanisms underlying the induction and regulation of CD103 in human iTregs and its downstream signaling pathways that may modulate lymphocyte effector function.

The current study examines the expression patterns of distinctive markers associated with memory Treg cells during the initial stages of iTreg differentiation, and unveils a particular subset of human CD103^+^ iTregs exhibiting immunosuppressive and immunoregulatory properties. These findings highlight the potential utility of iTregs in investigating molecules associated with Treg function.

### Limitations of the study

Here, human iTregs were generated in vitro from umbilical cord blood-derived naïve CD4^+^ T cells and characterized during the early stages of differentiation. However, the current study limits its investigation to healthy neonate cells at initial stages of Treg induction. While our study effectively demonstrates the immunosuppressive function of iTregs during the early stage of differentiation, it is acknowledged that the loss of FOXP3 expression over time and alterations in the epigenetic landscape can result in instability in iTreg cell identity and function. Questions to be addressed in the follow up studies include the changes in iTreg with time in culture and their tissue homing capabilities.

## Electronic supplementary material

Below is the link to the electronic supplementary material.

Supplementary Material 1

Supplementary Material 2

## Data Availability

The datasets used and/or analyzed in the present study are available from the corresponding author on reasonable request. The mass spectrometry proteomics raw and peak files have been deposited to ProteomeXchange via PRIDE [80], project accession ID: PXD052626.
