# Supplementary material for: Phenotypic profiling of human induced regulatory T cells at early differentiation: insights into distinct immunosuppressive potential
Source: Cell Mol Life Sci. 2024 Sep 12;81(1):399. doi: 10.1007/s00018-024-05429-3 (PMC11393232; doi:10.1007/s00018-024-05429-3)
Supplement: Supplementary file 1 — Supplementary Material 1 [file 18_2024_5429_MOESM1_ESM.pdf]

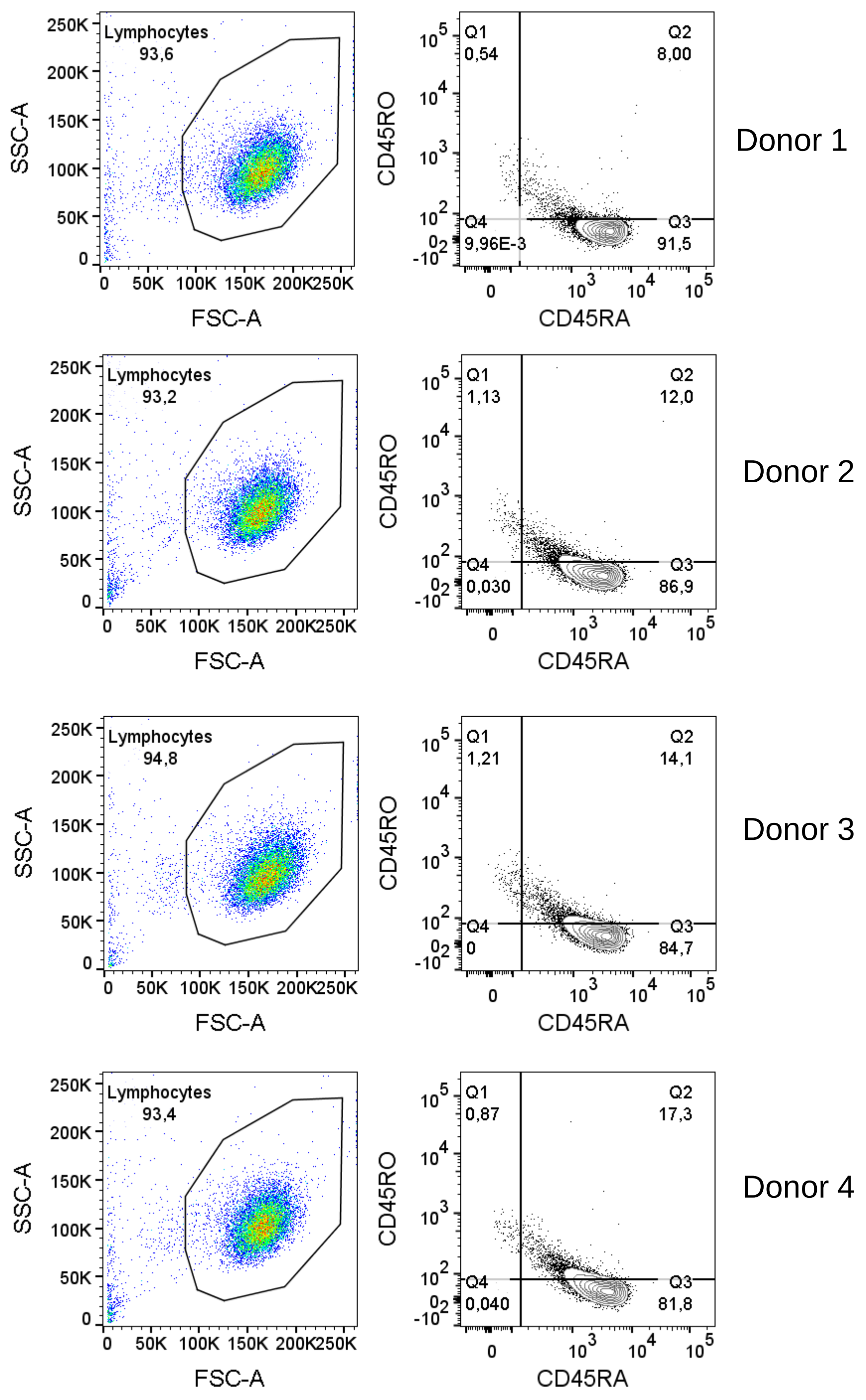

Figure S1. Surface staining of naïve $CD4^+$ T cells prior to CD25 depletion and iTreg induction. $CD4^+$ T cells were isolated from individual mononuclear cells (MNCs) using $CD4^+$ Dynal positive selection beads (Invitrogen) and stained with CD45RA (FITC-conjugated) and CD45RO (PE-conjugated) antibodies. Live lymphocytes were gated according to the FSC/SSC and plotted against CD45RA/CD45RO. Naïve $CD4^+$ T cells are shown in quadrant Q3 for four individual donors.

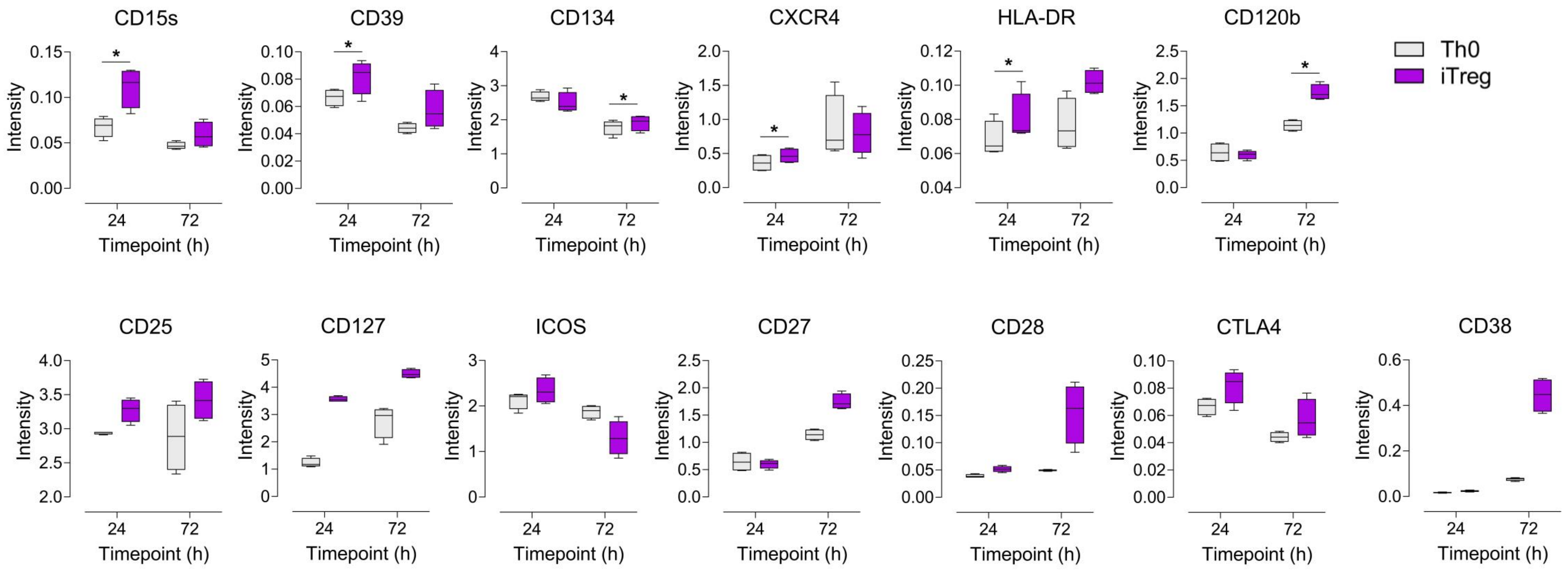

Figure S2. Marker expression profiles of iTreg and Th0 control cells at 24 and 72 h of differentiation are shown as box plots. Boxplot represents median and interquartile range, and whiskers extend to maximum and minimum values. Data are shown for four individual biological replicates Statistical significance was calculated using paired T-test (* FDR < 0.05).

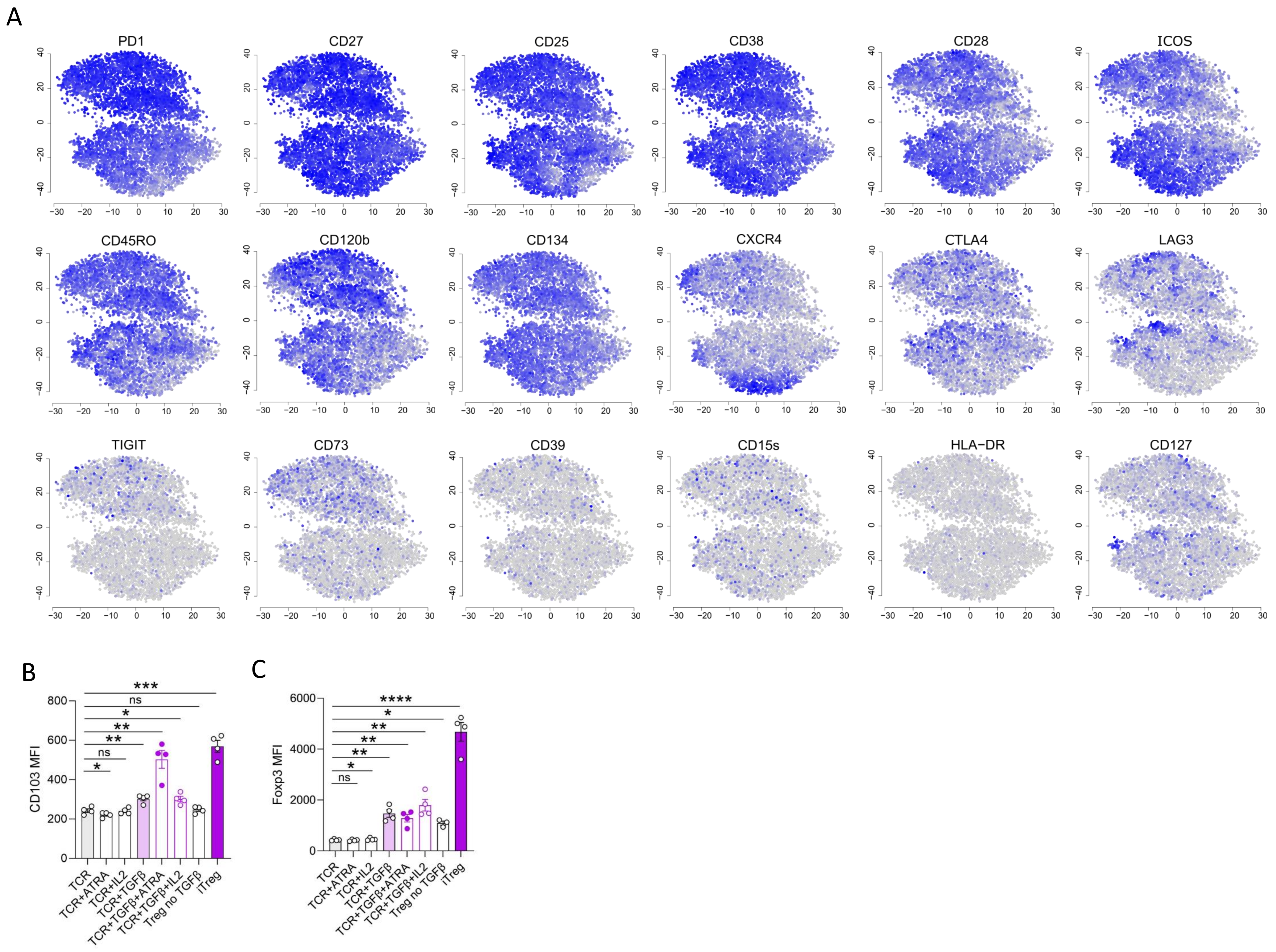

Figure S3. (A) The expression profiles of selected markers in iTreg and Th0 control cells at 72 h of differentiation are displayed as tSNE maps. (**B-C**) The mean fluorescence intensities (MFI) of CD103 surface and Foxp3 intracellular expression was determined by flow cytometry from naive $CD4^+$ T cells cultured for 72 h under activated Th0 condition, iTreg differentiation, or activated Th0 in the presence of IL2, ATRA and TGFβ alone or in combination. Data represent four biological replicates. Plots in **B** and **C** show mean ± SEM. Statistical significance is calculated using paired T-tests ($*p < 0.05$, $**p < 0.01$, $***p < 0.001$, $****p < 0.0001$).

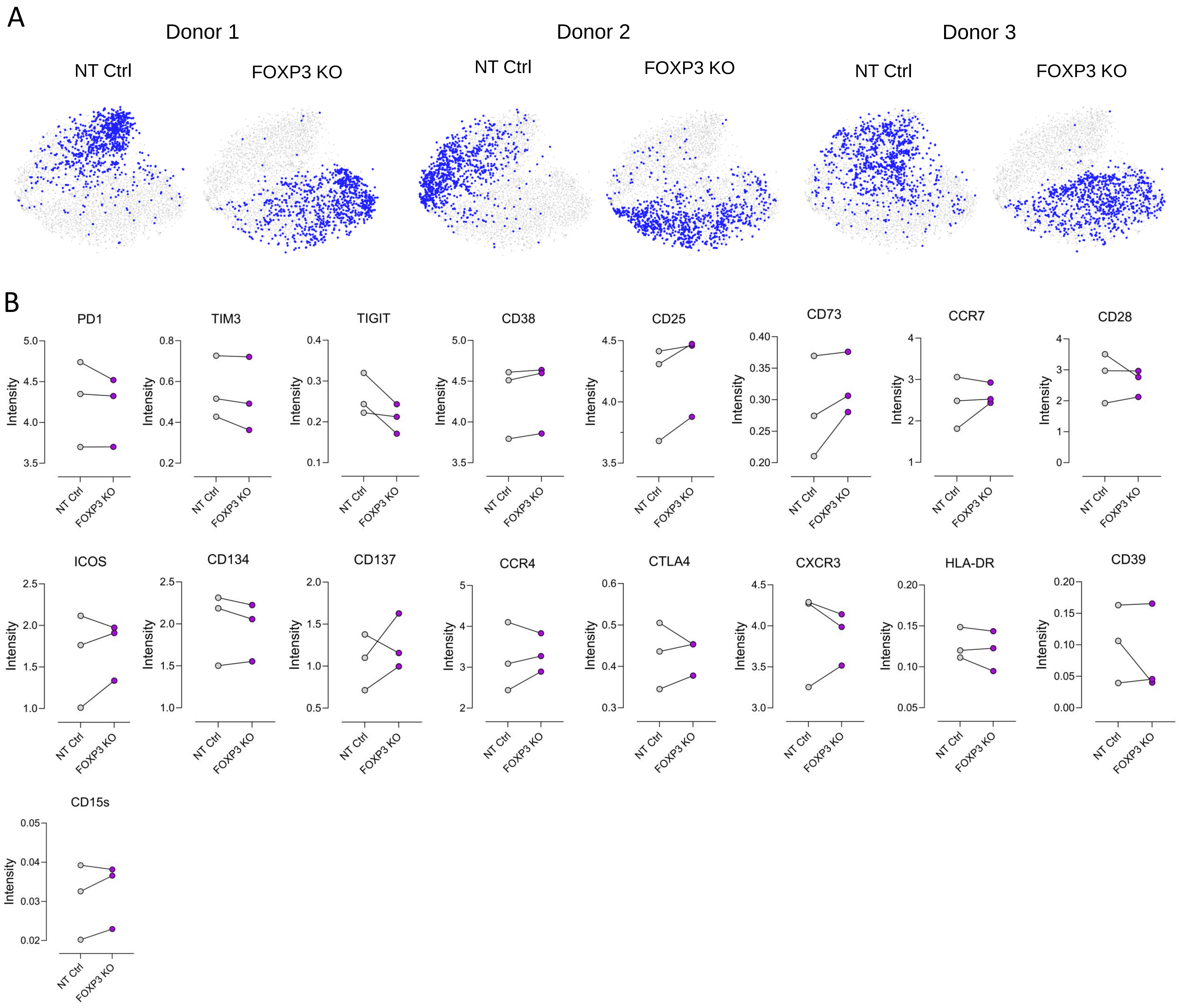

Figure S4 (**A**) A panel of 25 Treg-associated markers, was used to characterize FOXP3-ablated Tregs by mass cytometry. Marker intensity distribution of all markers combined for each donor are shown. (**B**) The intensity of markers expressed in NT Ctrl and Foxp3 KO iTregs at 72 h of differentiation are displayed as line plots for three individual donors. Data represent three biological replicates. Statistical significance is calculated using paired T-tests.

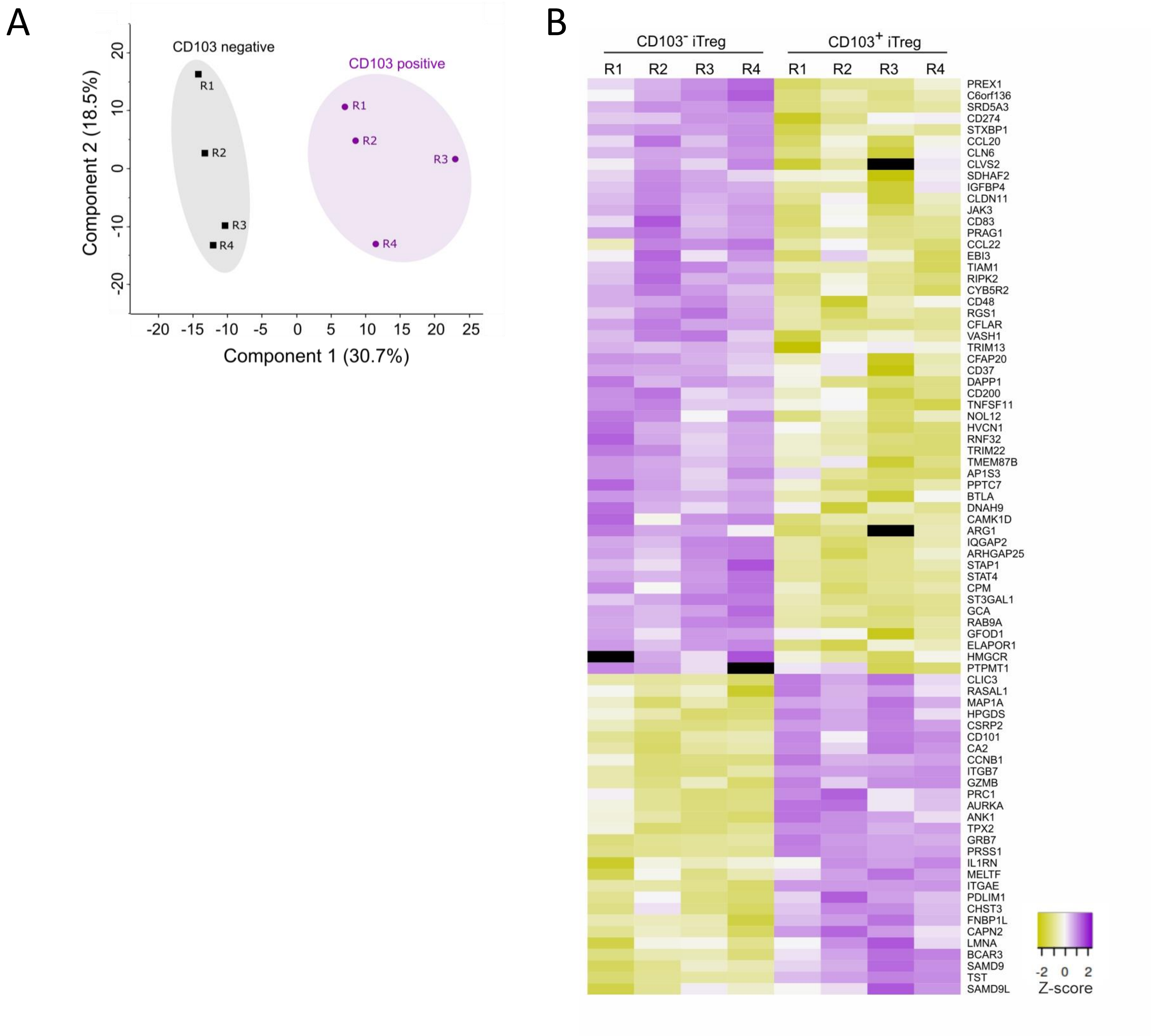

Figure S5. (**A**) Principal component analysis (PCA) revealed that CD103^{+} iTregs have a different protein profile than CD103^{-} iTregs. (**B**) Z score heatmaps standardized with a corrected p value of < 0.05 and log2FC of > 0.58 are shown for the top 80 significantly differentially expressed proteins of four biological replicates.
